# Notes on the sinistral helicoid snail *Bertia cambojiensis* (Reeve, 1860) from Vietnam (Eupulmonata, Dyakiidae)

**DOI:** 10.3897/zookeys.885.38980

**Published:** 2019-11-04

**Authors:** Chirasak Sutcharit, Fred Naggs, Jonathan Ablett, Pham Van Sang, Luong Van Hao, Somsak Panha

**Affiliations:** 1 Animal Systematics Research Unit, Department of Biology, Faculty of Science, Chulalongkorn University, Bangkok 10330, Thailand Chulalongkorn University Bangkok Thailand; 2 Mollusca Section, Invertebrates Division, Department of Life Sciences, The Natural History Museums, London SW7 5BD, UK The Natural History Museums London United Kingdom; 3 Vietnam National Museum of Nature (VNMN), Vietnam Academy of Science and Technology (VAST), 18 Hoang Quoc Viet St, Cau Giay, Hanoi, Vietnam Vietnam Academy of Science and Technology Hanoi Vietnam; 4 Center for Rescue and Conservation of Organism, Hoang Lien National Park, Vietnam Center for Rescue and Conservation of Organism Hoang Lien Vietnam

**Keywords:** Conservation, DNA barcoding, endangered, left-handed, systematics

## Abstract

Since the time of the original description there have been no precise locality records in Cambodia of *Bertia cambojiensis* (Reeve, 1860) and it was believed to be extinct. In 2012, a joint Natural History Museum survey with Vietnamese colleagues rediscovered living populations of this huge sinistral helicoid snail in a protected area of southern Vietnam. The genitalia and radula morphology are re-assessed and type specimens of all recognised congeners are figured herein. The unique morphological characters of this species are a small and simple penis, well-developed amatorial organ complex that incorporates four amatorial organ ducts, a short gametolytic organ complex and spiked papilla, and radula morphology with unicuspid teeth. The type locality of *B. cambojiensis*, which has been contentious, is determined here to be in the vicinity of ‘Brelum’, Vietnam, near the border with Cambodia. In addition, the nucleotide sequences of barcoding genes COI, 16SrRNA and 28S fragments were provided for further comparison.

## Introduction

The Dyakiidae Gude & Woodward, 1921, are a family of helicoid land snails restricted to Southeast Asia. The apomorphic characters of the family are the presence of an amatorial organ complex comprised of amatorial organ glands, amatorial organ ducts and amatorial papilla with a conchiolin spike (Godwin-Austen 1891, Laidlaw 1931, Baker 1941, Schileyko 2003). Of the 12 recognised genera in this family, three are sinistral: *Rhinocochlis* Thiele, 1931, endemic to Borneo; *Dyakia* Godwin-Austen, 1891, comprised of about 20 nominal species distributed in the Greater Sunda Islands and Peninsular Malaysia, and *Bertia* Ancey, 1887, endemic to eastern Indochina with three recognised species (Zilch 1959, Hausdorf 1995, Schileyko 2003, Sutcharit et al. 2012, Thach 2015).

Ancey (1887) described *Bertia* on the basis of the very large and sinistral, helicoid shell, initially as a monotypic genus based on *Helix cambojiensis* Reeve, 1860. Based on the shell characters, Thiele (1931: 1001) placed *Bertia* as a subgenus of *Ariophanta* Des Moulins, 1829, an Indian ariophantid genus that included large sinistral shells. The Bornean endemic species *Helix brookei* Adam & Reeve, 1848 was subsequently included in *Bertia* because of the very similar shell form (Baker 1941, Solem 1964). However, on the basis of the reproductive anatomy of *Helix brookei* (Godwin-Austen 1891, Weigmann 1898, Laidlaw 1932), Baker (1941) subsequently placed *Bertia* within the family Trochomorphidae. Baker placed *Helix brookei* in his new subgenus Bertia (Exrhysota). Zilch (1959) questioned the placement of *Exrhysota* in *Bertia*. Schileyko (2003) transferred *Bertia* to the Dyakiidae on the basis of characters of the genitalia and erected the new family Ryssotidae including *Exrhysota* as a distinct genus, which was subsequently synonymized with the Chronidae Thiele, 1931 by Bouchet et al. (2017).

*Bertia cambojiensis* (Reeve, 1860) was originally described under the name *Helix mouhoti* Reeve, 1860 [September], based on specimens collected by the famous French naturalist Henri Mouhot. Since this combination was a junior primary homonym of Pfeiffer (1860a [May]), it was replaced by *Helix cambojiensis* Reeve, 1860 [December] (Reeve 1860a, b). The type locality was given as “Cambojia” [= Cambodia]. Prior to Schileyko (2011), and for over 150 years following Reeve’s original description, no specific distribution records of *B. cambojiensis* were published. Schileyko (2003) figured and described the genital anatomy of a museum specimen identified as *B. cambojiensis* which was recorded as being from Cambodia but his figure of a shell was of a different specimen. *Bertia cambojiensis* was widely thought to be extinct (Abbott 1989, Coney 2001). However, examples were seen offered for sale on shell dealers websites based in China in 2014/2015 for over € 400; they are now listed for as low as € 20 but are currently unavailable.

Living populations of *B. cambojiensis* were discovered on a 2012 survey in Cat Tien National Park organized by the Vietnam National Museum of Nature (VNMN) and the Natural History Museum, London (NHM). *Bertia cambojiensis* were listed as Critically Endangered (CR) on the IUCN Red List of Threatened Species (2014) due to their apparent restricted distribution in lowland tropical forest patches. In addition to deforestation and habitat degradation, threats include use as food and as a traditional medicinal resource (Daniel 1869, Phong 2018). Furthermore, *B. cambojiensis* is considered to be at particular risk because it is highly sought after by shell collectors globally due to its strikingly attractive shell and perceived rarity. It may currently be locally abundant but is easily visible at night on the trunks of trees and extremely vulnerable to over-collection (Naggs 2014). To safeguard the survival of *B. cambojiensis*, captive breeding populations were set up at the Vietnamese National Museum of Nature and at the Zoological Society of London and viable cell preparations are stored at the NHM, London. In this study, we present new information on genitalia, anatomy and radula morphology of *B. cambojiensis* based on specimens in the NHM, London. Information on the type specimens of all recognised species in the genus are provided and the systematic position of “*Helix brookei*” is discussed.

## Materials and methods

### Samples

All voucher specimens deposited in the NHM, London were examined. Two preserved specimens in 70% ethanol (NHMUK 20130833 and 20130874) were dissected for examination of the genitalia, and radulae were extracted and examined under a scanning electron microscope (JEOL, JSM-5410 LV). The radula shape and teeth formula were observed and recorded. Cytochrome c oxidase subunit I (COI), 16S ribosomal RNA (16S) and 28S ribosomal RNA (28S) genes of *B. cambojiensis* samples were sequenced for DNA barcoding. For DNA extraction and PCR amplification conditions and amplified primers see Appendix 1.

### Abbreviations

**am** amatorial organ;

**amd** amatorial organ duct;

**amg** amatorial organ gland;

**amp** amatorial organ pilaster;

**at** atrium;

**e** epiphallus;

**fo** free oviduct;

**go** gametolytic organ (duct and sac);

**ov** oviduct;

**p** penis;

**pg** prostate gland;

**pp** penial pilaster;

**pr** penial retractor muscle;

**v** vagina;

**vd** vas deferens;

**vp** vaginal pilaster.

## Systematics

### Genus Bertia

Taxon classificationAnimaliaStylommatophoraDyakiidae

Ancey, 1887

5EE0ABFD-67EB-56EC-837E-193293F933D4

Bertia Ancey, 1887: 53. Baker 1941: 320, 321. Zilch 1959: 275, 276. Schileyko 2003: 1362.
Ariophanta (Bertia) Thiele, 1931: 1001.

#### Type species.

*Helix cambojiensis* Reeve, 1860, by original designation.

#### Remarks.

Thach (2015) recognised four nominal species in *Bertia*: “*Bertia*” *brookei* (Adam & Reeve, 1848), *B. cambojiensis* (type species), *B. pergrandis* (Smith, 1893) and *B. setzeri* Thach, 2015. Nevertheless, he had overlooked key characters of the genitalia published by Godwin-Austen (1891: pl. 6, fig. 6) and Schileyko (2003: 1345, 1346, fig. 1758). The Bornean endemic species, *Helix brookei* Adam & Reeve, 1848, exhibits a long and cylindrical gametolytic sac, lacks an epiphallus and amatorial organ complex, and a caudal foss and a caudal horn is absent; characters that unequivocally distinguish this species from the Dyakiidae (Godwin-Austen 1891, Laidlaw 1931, Sutcharit et al. 2012). It is clear that *Helix brookei* Adam & Reeve, 1848 does not belong in *Bertia* and we recognise the following three as *Bertia* species.

### Bertia cambojiensis

Taxon classificationAnimaliaStylommatophoraDyakiidae

(Reeve, 1860)

45F5B6E6-87BC-5CBB-83E2-99909A7EBF3D

Figs 1 , 2

Helix mouhoti Reeve, 1860a [Sep.]: 203, 204 [not Pfeiffer 1860a [May]: 136, pl. 50 fig. 5]. Type locality: Cambojia [Cambodia]. Pfeiffer 1860b: 173, 174, pl. 47, figs 1, 2. Pfeiffer 1868: 64.
Helix cambojiensis Reeve, 1860b [Dec.]: 455 [new replacement name]. Martens 1867: 76. Pfeiffer 1868: 64. Daniel 1869: 126–128. Morelet 1875: 250. Pfeiffer 1876: 78, 79. Pfeiffer and Kobelt 1881: 604, pl. 176, figs 1, 2. Tryon 1886: 18, pl. 6, fig. 9.
Helix cambodjensis : Mabille and le Mesle 1869: 128 [incorrect subsequent spelling].
Nanina cambodgiensis : Ancey 1887: 53 [incorrect subsequent spelling]. Fischer and Dautzenberg 1904: 393.
Ariophanta (Rhyssota) cambojiensis : Fischer 1891: 23.
Bertia cambodjiensis : Abbott 1989: 127 with text figure [incorrect subsequent spelling].
Bertia cambojiensis : Schileyko 2003: 1362, fig. 1777. Schileyko 2011: 37. Thach 2016: 142, fig. 271.

#### Material examined.

**Figure 1.**
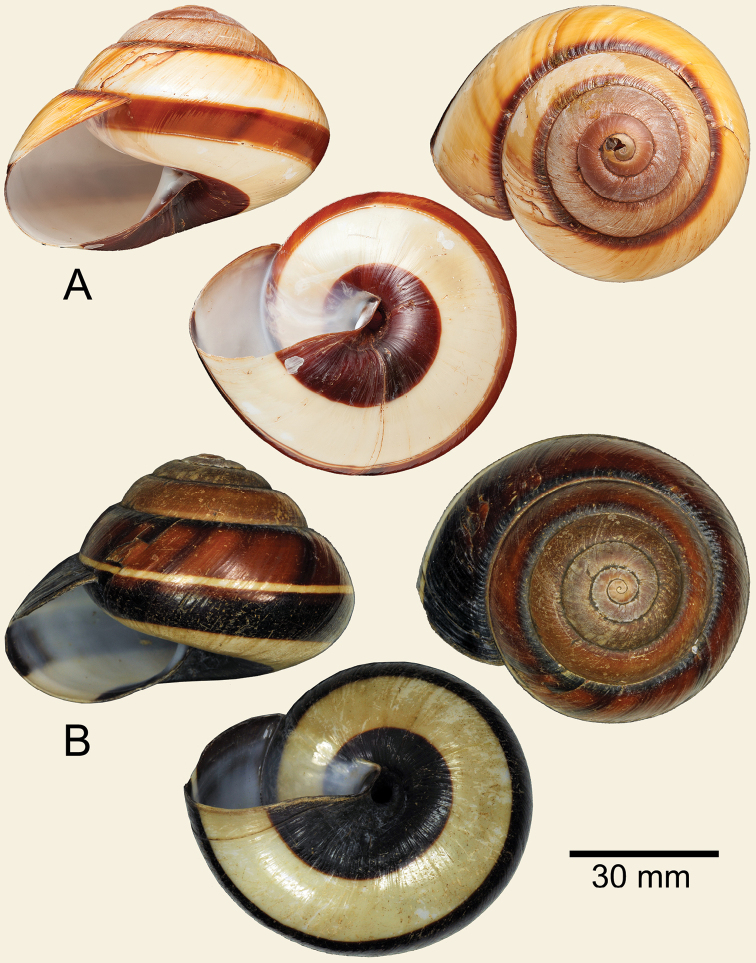
Shells of *Bertia cambojiensis***A** lectotype NHMUK 20130220 and **B** specimen NHMUK 20130833.

Two syntypes from the Cuming collection. The specimen figured by Pfeiffer (1860b: pl. 47, figs 1, 2) can be recognised by two broken growth lines; one on the border of penultimate and last whorls, and one on the last whorl close to the apertural lip (seen from umbilical view) and is here designated as the lectotype NMUK 20130220 (height 54.6 mm, width 76.2 mm; Fig. 1A). The remaining paralectotype NHMUK 20130219 measures height 54.4 mm and width 73.6 mm.

All additional specimens were from the area of Cat Tien National Park, Dong Nai Province, Vietnam (11°26.147"N, 107°25.643"E): NHMUK 20130818 from Lodge Gardens (4 specimens + 2 juveniles); NHMUK 20130833 from Cave Site (7 specimens + 1 juvenile; Fig. 1B; COI accession no. MN296022, 16sRNA accession no. MN296390); NHMUK 20130874 from Near Lake (6 specimens + 3 juveniles; COI accession no. MN296023, 16sRNA accession no. MN296391 and 28S accession no. MN296349). Measurements: shell height 49.9–55.7 mm, average 52.6 mm; shell width 64.4–74.5 mm, average 69.7 mm.

#### Description.

***Shell*.** Shell sinistral, large, dome shape and thickened. Whorls 6 to 7, increasing regularly, slightly convex, and with wide and shallow suture. Periostracum thin to slightly thickened, corneous. Spire convex, apex obtuse, embryonic shell large with smooth surface, following whorls possess a series of thin nodules on growth lines. Last whorl well rounded. Upper shell surface glossy, varying from rich cream and brownish to blackish bands; narrow pale white to yellowish spiral band on periphery; narrow reddish-brown to dark subsutural band. Below periphery always with reddish-brown to dark colour and with broad white spiral band surrounding umbilicus. Umbilical area usually reddish-brown or darker. Aperture ovate; parietal callus translucent whitish; columella thickened and slightly dilated. Lip simple or slightly thickened in old adults. Umbilicus narrowly perforate to rimate and deep.

**Figure 2.**
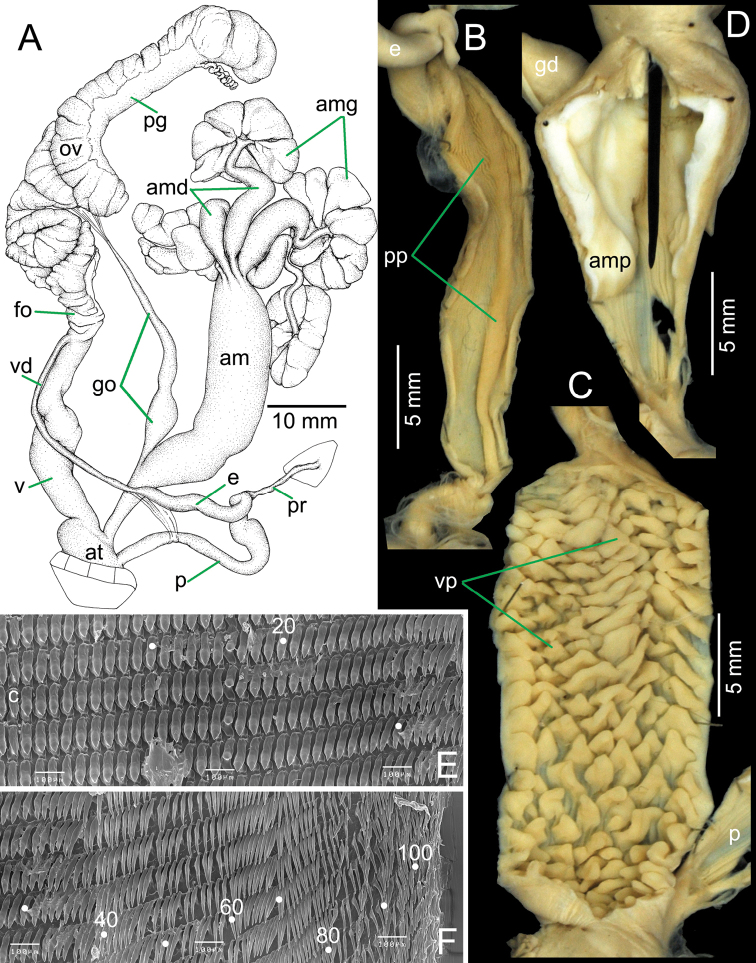
Genitalia and radula of *Bertia cambojiensis*, NHMUK 20130833. **A** overview of genital system **B–D** internal wall sculpture of **B** penis **C** amatorial organ and **D** vagina **E, F** SEM image of radula **E** central and inner latero-marginal teeth and **F** outermost teeth. Numbers indicate tooth order from lateral to marginal end; central tooth indicated by ‘C’.

***Genital organs.*** Atrium (at) very short (*N* = 2). Penis (p) long cylindrical tube. Penial retractor muscle (pr) short, thickened and attached distally to penis. Epiphallus (e) cylindrical tube, about half of penis length and slightly smaller diameter than distal penis. Vas deferens (vd) relatively smaller diameter and thin tube extending from free oviduct (fo) and entering epiphallus, apically; flagellum absent (Fig. 2A). Internal wall of penis with large penial pilasters (pp) for nearly entire length of chamber; proximally with smooth surface and distally with very thin crenellations on surface; penial verge absent (Fig. 2B).

Gametolytic organ (go; duct and sac undifferentiated) proximal to genital opening about one-fourth of amatorial organ length. Slightly swollen proximally, then tapering to small, long cylindrical tube and attached to ovary with thin connective tissues. Amatorial organ (am) well-developed enlarged cylinder; proximally attached to atrium. Amatorial organ glands (amg) enlarged, composed distally of four major lobes bounded to amatorial organ by thin connective tissue. Each of four major lobes of the amatorial organ gland extend proximally into thick amatorial organ ducts (amd) that are twisted together and bound with thin connective tissue before entering the distal tip of the amatorial organ (Fig. 2A). Internal wall sculpture of amatorial organ: proximally smooth surface for about one-third of chamber; distally consists of smooth surface of enlarged longitudinal amatorial organ pilasters (amp). Amatorial organ papilla small, short and conical, tipped by a large and long blackish spike (Fig. 2C).

Vagina (v) long enlarged cylinder, about the same length as penis. Free oviduct (fo) cylindrical tube; oviduct (ov) long with lobules; prostate gland (pg) bound to oviduct. Most of albumen gland, hermaphroditic duct and gland missing from figured specimen (Fig. 2A). Internal wall of vagina sculptured with uniform scale-like or triangular lingulate pilasters (vp), varying in size from small to large (Fig. 2D).

***Radula*.** Teeth arranged in wide angle V-shaped rows with approximately 245 teeth with formula ((124-118)-1-(120-122)). Central tooth symmetric unicuspid and triangular. Lateral and marginal teeth undifferentiated, slightly curved unicuspid, triangular, and inclined towards central tooth (Fig. 2E). Inner teeth similar in shape and size to central teeth and then gradually reducing to slender and elongate sword-shaped with pointed cusp; outermost teeth slightly shorter with pointed tip (Fig. 2F).

***External features*.** Living snail with long, blackish-brown tentacles. Skin reticulated brown with black reticulations around head. Foot sole relatively elongated, broad and unipartite. Sole of foot brownish to orange and unspotted; side of body brownish. Tail curved mid-dorsally, tall dome-shaped in cross section. Caudal horn not overhanging; caudal foss long vertical slit in tail above sole margin. Typical aulacopoda with well-defined pedal groove.

Mantle collar large and shell lobes thickened, shell lappet absent. Right dorsal lobe (right side of anus) large and thickened. Left dorsal lobe (left side of anus) composed of thick crescentric anterior left dorsal lobe and thin elongated posterior left dorsal lobe. Pulmonary cavity typically sigmurethran.

#### Distribution.

The range of *B. cambojiensis* is likely to be confined to localised forest patches within Dong Nai, Binh Thuan, Lam Dong and Binh Phuc provinces. Records far outside of this range such as Ba Vi National Park (Schileyko 2011) are very doubtful and to date, no specific localities have been recorded for Cambodia.

#### Remarks.

The specimens from southern Vietnam that we examined exhibit clear differences in genital anatomy from those described by Schileyko (2003: 1362, fig. 1777b, c) that were based on a specimen in the Muséum National ďHistoire Naturelle, Paris, recorded from Cambodia. The Paris specimen exhibits a long, cylindrical gametolytic duct (spermathecal stalk in Schileyko) and a bulbous gametolytic sac (receptaculum seminalis in Schileyko), distally pointed. The amatorial organ contains numerous amatorial organ ducts. In the NHM specimens the gametolytic duct is short and the amatorial organ includes four amatorial organ ducts. On the basis of numerous studies in the reproductive anatomy of pulmonate snails, these character differences strongly suggest species level distinction. The shell of the Paris specimen was not figured by Schileyko, his figured shell of *B. cambojiensis* (fig. 1777a) being from the Naturalis Museum, Leiden, which was also recorded as being from Cambodia.

In December 1858, the French explorer and naturalist Henri Mouhot set off from Bangkok by sea to the port of Komput (Kampot), Cambodia, from where he continued on his destination to Brelum in Annam (Vietnam), He reached the Catholic Mission in Brelum in August 1859. Stuart et al. (2006) state that Brelum was considered to be within the confines of Cambodia at the time of Mouhot’s visit. However, Mouhot (1864a: 237) unequivocally described how ‘It took us two long days’ journey to reach Brelum’ after having passed the Cambodian border town of Pump-Ka-Daye. Brelum was Mouhot’s collecting base for the following three months before setting off on his return journey in November (Mouhot 1864a). During his stay in Brelum his host, the missionary R.P. Guillon, wanted Mouhot to sample the local escargot, which proved to be *B. cambojiensis*. Staggered by the beauty of this snail Mouhot set about obtaining examples for his collections from the local area, where it was abundant (Daniel 1869). Daniel (1869) went on to state that *B. cambojiensis* had not been found in Cambodia. Historical records may all relate to the mistaken assumption that the type locality was Cambodia. Mouhot was dependant on the sale of his collections to finance his fieldwork and it is likely that he shipped a large number of specimens of *B. cambojiensis* to his agent in London, Samuel Stevens, who would have sold them on to museums and collectors throughout Europe, presumably labelled with the locality ‘Cambodia’. The field and travel conditions impacted severely on Mouhot’s collections and he clearly had insufficient time to arrange them in good order before they were shipped. Writing to Stevens from Pinhalu, near Phnom Penh, on 20^th^ December, 1859 Mouhot (1864b: 248) commented: ‘…I have little time to give you any details as to what I despatch from Komput and Singapore’.

According to Ashburton (1864), the location of Brelum, the type locality, is 11°58"N, 107°12"E, which is at an altitude of 460 m (Google Earth). This location is 30 km north of the north-western boundary of Cat Tien National Park and some 94 km north and west of the entrance to Cat Tien National Park. However, there remains some doubt as to the exact location of Brelum. Under the heading of a letter to Stevens, Mouhot (1864b: 241) stated: ‘Brelum, among the savage Stiêns, lat, 11°46'30"N, 103°3'W merid. of Paris, 15^th^ October, 1859’. This is clearly wrong because in addition to W (west) it should be E (east) and in the following text he gives exactly the same geo-reference for Pinhalu that he states to be about nine miles from the capital, Phnom Penh 11°46'58"N, 104°22'59"E (Google Earth) whereas Mouhot’s reading 11°46'30"N, 103°3'W Paris (= 2°20'14.03" east of Greenwich) = 105°23'14"E, is some 54 km to the east and north of Phnom Penh (Google Earth). Kottelat and Tan (2017) identify Bro Lam Phe, 11°56'N, 106°40'E, in Loc Ninh District, Binh Phuoc Province, Vietnam, as the location of Brelum. This places Brelum at 100 m altitude and only 2 km from the closest boundary with Cambodia, which at this point follows the course of a meandering tributary of the Mekong. It is some 70 km from the boundary of Cat Tien National Park and 100 km from the main park entrance. It seems that Mouhot would have been most unlikely to retrace his steps and cross the river into Cambodia before his return journey and we conclude that he only collected *B. cambojiensis* from the vicinity of Brelum in Vietnam. Nevertheless, being in close proximity to the Cambodian border it does seem likely that *B. cambojiensis* will occur in this area of Cambodia.

### Bertia pergrandis

Taxon classificationAnimaliaStylommatophoraDyakiidae

(Smith, 1893)

0D1FD19D-56BD-5C1E-B33E-FF5F8B445E76

Fig. 3A

Rhyssota pergrandis Smith, 1893: 11, with text figure. Type locality: Annam.
Nanina pergrandis : Fischer and Dautzenberg 1904: 4.
Ariophanta pergrandis : Schileyko 2011: 29.
Bertia pergrandis : Thach 2015: 214, figs 9–12. Thach 2016: 62, pl. 18, fig. 270, pl. 19, fig. 272.

#### Material examined.

**Figure 3.**
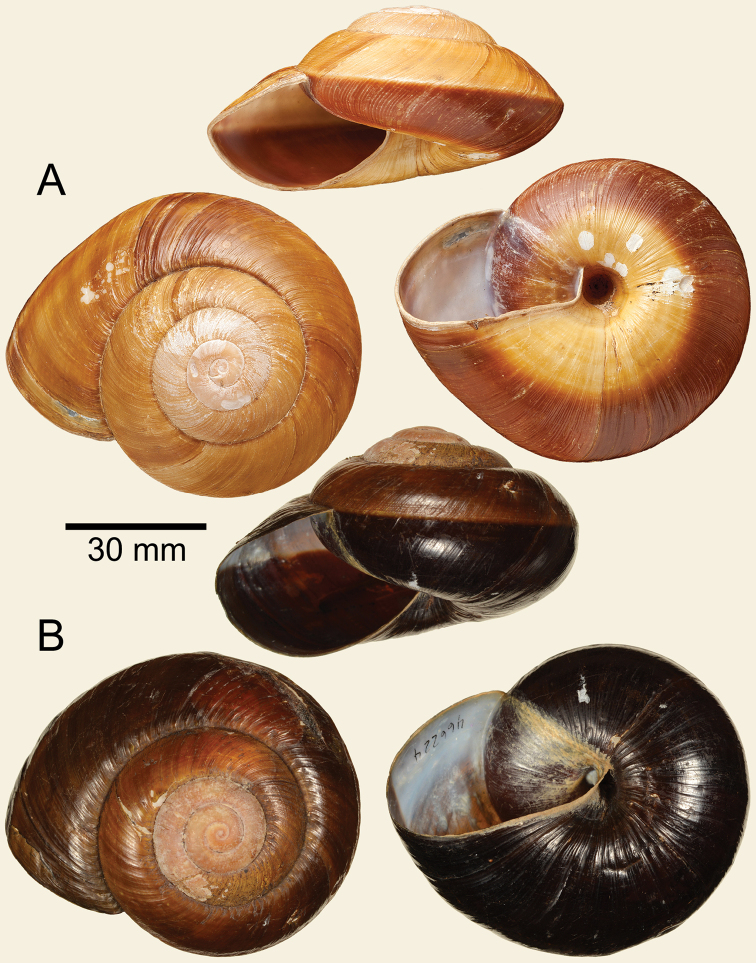
**A***Bertia pergrandis*, syntype NHMUK 1893.2.26.1 **B***Bertia setzeri*, holotype ANSP 466244.

Syntype NHMUK 1893.2.26.1 (1 shell, Fig. 3A).

#### Remarks.

*Ariophanta* ranges from India to Indochina (Godwin-Austen 1888, Schileyko 2003) and includes large species with both dextral and sinistral shells. Although the reproductive anatomy of *Ariophanta* and *Bertia* are quite distinct the sinistral shells exhibit a close convergence in shell form and definitive generic attribution requires investigation of reproductive anatomy or molecular evidence. We provisionally follow Thach (2015) in attributing this species to *Bertia* based on its very close similarity to the type species in shell form and colour and to the presence of earlier whorls with nodules arranged along the growth lines. *Bertia pergrandis* differs from the type and following species in possessing a stronger peripheral keel and widely open umbilicus; the other species exhibit a rounded last whorl and rimate umbilicus.

### Bertia setzeri

Taxon classificationAnimaliaStylommatophoraDyakiidae

Thach, 2015

55CB27B4-320B-58BB-A7D1-AD1706EC1A5B

Fig. 3B

Bertia setzeri Thach, 2015: 240, 241, figs 1–4, 17–20. Type locality: Khanh Vinh District and Nha Trang outskirts, Khanh Hoa Province, central Vietnam. Thach 2016: 62, pl. 18, figs 267–269.

#### Remark.

Images of the holotype are shown in Figure 3B. Thach (2015) mentioned that this species differs from the *B. cambojiensis* in possessing a more depressed shell, slightly angular periphery and a monochrome dark colour below the periphery. The living specimen figured by Thach (2015: figs 17, 19) shows a similar aulacopod foot sole with less developed caudal horn than in the type species. An examination of the reproductive organs or molecular data are required to determine its systematic status.

## Supplementary Material

XML Treatment for Bertia

XML Treatment for Bertia cambojiensis

XML Treatment for Bertia pergrandis

XML Treatment for Bertia setzeri

## Appendix 1

**Molecular methods**

DNA was extracted from small pieces of mantle collar tissue using a NucleoSpin Tissue kit (MACHEREY-NAGEL), following the manufacturer’s protocol. The COI gene was amplified using the universal primers LCO1490 (5'-GGTCAACAAATCATAAAGATATTGG-3’) and HCO2198 (5'-TAAACTTCAGGGTGACCAAAAAAT-3’) (Folmer et al. 1994). The 16S gene was amplified using the primers 16Sar (5'-CGCCTGTTTATCAAAAACAT-3') and 16Sbr (5'-CCGGTCTGAACTCAGATCACGT-3’) (Palumbi et al. 1991). The 28S gene was amplified using the primers 28SF4 (5’-AGTACCGTGAGGGAAAGTTG-3’) and 28SR5 (5’-ACGGGACGGGCCGGTGGTGC-3’) (Morgan et al. 2002). The thermal cycling was performed at 94 °C for 3 min, followed by 35 cycles of 94 °C for 30 s, 42–52 °C (depending on samples and gene) for 60 s, extension at 72 °C for 90 s, then followed by a final 72 °C for 5 min. All PCR products were bi-directional sequenced by an automated ABI prism 3730XL sequencer at Bio Basic Inc., Canada, with the same PCR primers. The resulting nucleotide sequences of COI, 16SrRNA and 28S fragments are 640 bp, 456 bp and 536 bp long, respectively. Nucleotide sequences and deposited in the GenBank database with accession numbers: MN296022–3 for COI, MN296390–1 for 16S and MN296349 for 28S.

## References

[B1] Abbott RT (1989) Compendium of Landshells. American Malacologists Inc., Melbourne, Florida, 240 pp.

[B2] Adam A Reeve L (1848) Zoology: Mollusca. In: Belcher E (ED) The Zoology of the Voyage of H.M.S. Samarang, Under the Command of Captain Sir Edward Belcher, C.B., F.R.A.S., F.G.S., During the Years 1843–1846, 1–87. 10.5962/bhl.title.120176

[B3] Ancey C-F (1887) Description of new genera or subgenera of Helicidae. The Conchologist Exchange 1: 53, 54.

[B4] Ashburton J (1864) Paper read at the Royal Geographical Society 10^th^ March, 1862. In: Mouhot H (Ed.) Travels in the Central Parts of Indo-China (Siam), Cambodia, and Laos, During the Years 1858, 1859, and 1860.Murray, London, Volume 2: 296–300.

[B5] Baker HB (1941) Zonitid snails from Pacific Islands, Part 3. Genera other the Microcystinae. Bernice P. Bishop Museum Bulletin 166: 205–346.

[B6] Bouchet P Rocroi J-P Hausdorf B Kaim A Kano Y Nützel A Parkhaev P Schrödl M Strong EE (2017) Revised classification, nomenclator and typification of gastropod and monoplacophoran families. Malacologia 61: 1–526. 10.4002/040.061.0201

[B7] Coney C (2001) Snails. In: Hildyard A (Ed.) Endangered Wildlife and Plants of the World.Marshall Cavendish, 1336–1343.

[B8] Daniel F (1869) Note sur la provenance exacte de ľ *Helix cambojiensis*, Reeve. Journal de Conchyliologie 17: 126–128.

[B9] Des Moulins MC (1829) Notice sur un Limaçon de la côte de Malabar, observé vivant à Bordeaux. Bulletin ďHistoire Naturelle de la Société Linnéenne de Bordeaux 3: 227–238.

[B10] Fischer H Dautzenberg P (1904) Catalogue des mollusques terrestres et fluviatiles de ľIndo-Chine orientale cites jusqu’ à ce jour. In: Mission Pavie, Etudes diverses, Volume 3: 390–450.

[B11] Fischer P (1891) Catalogue et distribution gèographique des Mollusques terrestres, fluviatilies & marins ďune partie de ľIndo-Chine (Siam, Laos, Cambodge, Cochinchine, Annam, Tonkin). Imprimerie Dejussieu Père et Fils, Autun, 192 pp 10.5962/bhl.title.14809

[B12] Folmer O Black M Hoeh W Lutz R Vrijenhoek R (1994) DNA primers for amplification of mitochondrial cytochrome c oxidase subunit I from diverse metazoan invertebrates. Molecular Marine Biology and Biotechnology 3: 294–299. 7881515

[B13] Godwin-Austen HH (1888 [1882–1888]) Land and Freshwater Mollusca of India, including South Arabia, Baluchistan, Afghanistan, Kashmir, Nepal, Burmah, Pegu, Tenasserim, Malay Peninsula, Ceylon, and other islands of the Indian Ocean. Supplementary to Messrs. Theobald and Hanley’s Conchologia Indica, Taylor & Francis, London. Volumes 1: 1–157, pls 1–62. [Part 6: pp. 207–257, pls. 52–62]

[B14] Godwin-Austen HH (1891) On a collection of land shells made in Borneo by Mr. Everett, with descriptions of supposed new species. Part II. Zonitidae and Helicinidae. Proceedings of the Zoological Society of London 59: 22–47. 10.1111/j.1469-7998.1891.tb06805.x

[B15] Gude GK Woodward BB (1921) On *Helicella* Férussac. Proceedings of the Malacological Society of London 14: 174–190.

[B16] Hausdorf B (1995) A preliminary phylogenetics and biogeographic analysis of Dyakiidae (Gastropoda: Stylommatophora) and a biogeographic analysis of other Sundaland taxa. Cladistics 11: 359–376. 10.1111/j.1096-0031.1995.tb00095.x 34920646

[B17] Johnson RI (1969) Pfeiffer’s Novitates Conchologicae, Series I, Land Mollusca, 1854–1879, and Dunker’s Novitates Conchologicae, Series II, Marine Mollusca, 1862–1882. A complete collation. Journal of the Society for Bibliography of Natural History 5: 236–239. 10.3366/jsbnh.1969.5.3.236

[B18] Kottelat M Tan HH (2017) Three new species of archerfishes from the freshwaters of Southeast Asia (Teleostei: Toxotidae) and notes on Henri Mouhot’s fish collections. Ichthyological Exploration of Freshwaters 952: 1–19.

[B19] Laidlaw FF (1931) On a new subfamily Dyakiinae of the Zonitidae. Proceedings of the Malacological Society of London 19: 190–201.

[B20] Laidlaw FF (1932) Notes on Ariophantidae from the Malay Peninsula, with description of new genera. Proceedings of the Malacological Society of London 18: 80–84.

[B21] Mabille J le Mesle G (1869) Observation sur la fanue malacologique de la Cochinchine et du Cambodje, comprenant la description des espèces nouvelles. Journal de Conchyliologie 14: 117–138.

[B22] Martens E von (1867) Die Landschnecken. Die Preussische Expedition nach Ost-Asien. Zoologischer Teil, II; Königliche Geheime Ober-Hofdruckerei, Berlin, 477 pp.

[B23] Morelet A (1875) Series Conchyliologiques, comprenant Ľénumération de mollusques terrestres et fluviatites, recueillis pendant le cours de différents voyages, ainsi que la description, de plusieurs espèces nouvelles. IV. 4e livraison Indo Chine, 227–377.

[B24] Morgan JA De Jong RJ Jung Y Khallaayoune K Kock S Mkoji GM Loker ES (2002) A phylogeny of planorbid snails, with implications for the evolution of *Schistosoma parasites*. Molecular Phylogenetics and Evolution 25: 477–488. 10.1016/S1055-7903(02)00280-4 12450752

[B25] Mouhot H (1864a) Travels in the central parts of Indo-China (Siam), Cambodia, and Laos, during the years 1858, 1859, and 1860. Murray, London, Volume 1, 303 pp.

[B26] Mouhot H (1864b) Travels in the central parts of Indo-China (Siam), Cambodia, and Laos, during the years 1858, 1859, and 1860. Murray, London, Volume 2, 301 pp.

[B27] Naggs F (2014) *Bertia cambojiensis* The IUCN Red List of Threatened Species. Version 2014.3. www.iucnredlist.org [Downloaded on 24 April 2015]

[B28] Palumbi S Martin A Romano S McMillan WO Stice L Grabowski G (1991) The Simple Fool’s Guide to PCR Version 2.0. Honolulu, HI: Department of Zoology and Kewalo Marine Laboratory, University of Hawaii.

[B29] Pfeiffer L (1860a) Descriptions of thirty-six new species of land-shells from Mr. H. Cuming’s collection. Proceedings of the Zoological Society of London 28: 133–141.

[B30] Pfeiffer L (1860b [1860–1866]) Novitates Conchologicae. Series Prima. Mollusca Extramarina. Band 2. Verlag von Theodor Fischer, Cassel, 139–303. [pls 37–72. p. 139–160, pls 37–42 (1860] [Published in parts, dates follow Johnson (1969)]

[B31] Pfeiffer L (1868) Monographia Heliceorum Viventum, Supplement Tertium. Volume 5. F.A. Brockhaus, Lipsiae, 565 pp.

[B32] Pfeiffer L (1876) Monographia Heliceorum Viventum, Supplement Tertium. Volume 7. F.A. Brockhaus, Lipsiae, 674 pp.

[B33] Pfeiffer L Kobelt W (1881 [1877–1897]) Die Schnirkelschnecken nebst den zunächst verwandten Gattungen. In: Systematisches Conchylien-Cabinet von Martini und Chemnitz, Ersten Bandes, zwölfte Abtheilung, vierter Theil 1(12) (4): 595–610. [pls. 173–176]

[B34] Phong TH (2018) Distribution and conservation of the endangered giant land snail (*Bertia cambojiensis*) in Southern Vietnam. In: Proceeding of Rufford Small Grants Conference. Institute of Ecology and Biological Resources, Vietnam Academy of Science and Technology, Hanoi, Vietnam 18, 19 pp [19^th^ – 20^th^ October, 2018]

[B35] Reeve L (1860a) On two new species of shells from Cambojia. Annals and Magazine of Natural History, Series 3(6): 203–204. 10.1080/00222936008697308

[B36] Reeve L (1860b) *Helix mouhoti*. Annals and Magazine of Natural History, Series 3(6): 455 10.1080/00222936008697366

[B37] Schileyko AA (2003) Treatise on recent terrestrial pulmonated mollusks. Ariophantidae, Ostracolethaidae, Ryssotidae, Milacidae, Dyakiidae, Staffordiidae, Gastrodontidae, Zonitidae, Daudebardiidae, Parmacellidae. Ruthenica, Supplement 2, Part 10: 1309–1466.

[B38] Schileyko AA (2011) Check-list of land pulmonate molluscs of Vietnam (Gastropoda: Stylommatophora). Ruthenica 21: 1–68.

[B39] Smith EA (1893) Descriptions of six new species of land shells from Annam. Proceedings of the Malacological Society of London 1: 10–13.

[B40] Solem A (1964) A collection of non-marine mollusks from Sabah. Sabah Society Journal 11: 1–40.

[B41] Stuart BL Sok K Neang T (2006) A collection of amphibians and reptiles from hilly eastern Cambodia. Raffles Bulletin of Zoology 54: 129–155.

[B42] Sutcharit C Tongkerd P Tan AS-H Panha S (2012) Taxonomic revision of *Dyakia janus* from Peninsular Malaysia (Pulmonata: Dyakiidae), with notes on other sinistrally coiled helicarionids. The Raffles Bulletin of Zoology 60: 279–287.

[B43] Thach NN (2015) *Bertia setzeri*, a new species of land snail from Vietnam (Gastropoda: Stylommatophora: Dyakiidae). The Festivus 47: 240–242.

[B44] Thach NN (2016) Vietnamese new mollusks with 59 new species. 48HrBooks Company, Ohio, 205 pp.

[B45] Thiele J (1931 [1929–1935]) Handbuch der Systematischen Weichtierkunde. Gustav Fischer, Jena, 1134 pp. [p. 1–376 (1929); p. 377–778 (1931); p. 779–1022 (1934); p. 1023–1134 (1935)]

[B46] Tryon Jr GW (1886) Manual of Conchology, Structure and Systematic, with Illustrations of the Species, 2^nd^ Series, Volume 2. Academy of Natural Science Philadelphia, Philadelphia, 265 pp.

[B47] Weigmann F (1898) Ergebnisse einer zoologischen forschungsreise in den Molukken und Borneo, landmollusken (Stylommatophoren). Zootomischer Teil Abhandlungen Senckenbergischen Naturforschenden Gesellschaft, 24(Hft. 3): 289–557. [pls 21–31]

[B48] Zilch A (1959) Gastropoda, Euthyneura. In: Schindewolf OH (Ed.) Handbuch der Paläozoologie, Band 6, Gastropoda.Gebrüder Borntraeger, Berlin, 1–400.

